# Combination therapy with TNF inhibitors plus biologics targeting type 2 inflammatory conditions in patients with rheumatoid arthritis: a case series

**DOI:** 10.1093/rap/rkaf150

**Published:** 2025-12-27

**Authors:** Anthony J Ocon, Azza Abdalla, Ranjini Vengilote, Allison Ramsey, S Shahzad Mustafa

**Affiliations:** Division of Allergy, Immunology, and Rheumatology, Rochester Regional Health, Rochester, NY, USA; Division of Allergy, Immunology, and Rheumatology, University of Rochester Medical School, Rochester, NY, USA; Department of Internal Medicine, Rochester Regional Health, Rochester, NY, USA; Department of Internal Medicine, Rochester Regional Health, Rochester, NY, USA; Division of Allergy, Immunology, and Rheumatology, Rochester Regional Health, Rochester, NY, USA; Division of Allergy, Immunology, and Rheumatology, University of Rochester Medical School, Rochester, NY, USA; Division of Allergy, Immunology, and Rheumatology, Rochester Regional Health, Rochester, NY, USA; Division of Allergy, Immunology, and Rheumatology, University of Rochester Medical School, Rochester, NY, USA

**Keywords:** rheumatoid arthritis, asthma, atopic dermatitis, urticaria, TNF inhibitor, type 2 inflammation

## Abstract

**Objectives:**

Patients with RA treated with TNF inhibitors (TNFis) may experience type 2 inflammatory conditions such as asthma, atopic dermatitis or urticaria. Multiple biologic agents targeting type 2 inflammation are available. Combination biologic therapy targeting types 1 and 2 inflammation is not well described. We present a series of patients on a combination TNFi and biologic agent targeting type 2 inflammation.

**Methods:**

A retrospective case series of RA patients on TNFi receiving a biologic agent for type 2 inflammatory conditions was compiled. Descriptive data, duration of biologic use, incident bacterial infections and corticosteroid used 6 months before and after combination biologic agent use was collected.

**Results:**

Twelve patients were included. The mean overlap of combination biologic therapy was 83.7 weeks (95% CI 56.0, 111.4) and the median was 92.6 weeks [interquartile range (IQR) 36.4–109.8]. The mean corticosteroid cumulative dose 6 months prior to dual biologic agents was 463 mg prednisone equivalent (95% CI 131, 795) and the median was 265 mg (IQR 75–570). The mean corticosteroid cumulative dose 6 months after dual biologic agents was 241 mg prednisone equivalent (95% CI −21, 503) and the median was 0 mg (IQR 0–275). Six bacterial infections occurred prior to combination biologic therapy compared with five after initiating dual biologics.

**Conclusion:**

This case series demonstrates that adding a second biologic agent to target type 2 inflammatory conditions in RA patients on TNFi did not increase incident bacterial infections and may decrease corticosteroid use.

Key messages

- Rheumatoid arthritis patients on TNF inhibitors may use a second biologic agent targeting type 2 inflammation for asthma, atopic dermatitis or urticaria.
- Dual biologic use was not associated with an increase in infections.
- Dual biologic use may decrease corticosteroid use.

## Introduction

Patients with RA are commonly treated with immunosuppressive biologic agents, often with TNF inhibitors (TNFis). Although TNFis are efficacious at modulating type 1 inflammation in RA, increased risk of infection is a known side effect [1]. Patients with RA may also experience type 2 inflammatory conditions such as asthma, atopic dermatitis (AD) or chronic spontaneous urticaria (CSU). Multiple biologic agents that target type 2 inflammation are approved for the management of these conditions, including omalizumab (an IgG1 anti-IgE monoclonal antibody that selectively binds to the C-ε-3 locus of IgE, preventing interaction with Fc-ε-RI), mepolizumab (an IgG1 anti-IL-5 monoclonal antibody that selectively binds to the α-chain of the IL-5 receptor), dupilumab (an IgG4 monoclonal antibody that selectively binds to the IL-4 receptor α subunit inhibiting IL‐4 and IL‐13 signalling), benralizumab (an IgG1 anti-IL5-Rα monoclonal antibody that binds to IL-5Rα as well as induces antibody-directed cell-mediated cytotoxicity of eosinophils and basophils) and tezepelumab (an IgG2 monoclonal antibody binding to thymic stromal lymphopoietin). There is minimal published literature on combination biologic therapy, including separate agents targeting type 1 and type 2 inflammation. A meta-analysis of >600 patients with RA revealed therapy with dual biologics targeting type 1 inflammation significantly increased the risk of serious infection [2]. However, combination biologic therapy separately targeting type 1 and type 2 inflammation may not confer this increased risk of infection. Malik *et al.* [3] presented a series of three patients with rheumatic disease treated with TNFi in combination with asthma biologics, with none experiencing any serious adverse events, including infection. Given the paucity of data on dual biologic therapy in the setting of rheumatologic and type 2 inflammatory conditions, we present a case series of patients with RA treated with TNFi and biologics targeting type 2 inflammation.

## Methods

Patients were identified from specialty pharmacy records using proprietary software (CPS Solutions, Dublin, OH, USA) from 1 January 2018 to 30 June 2024. A retrospective electronic health record (Epic, Verona, WI, USA) chart review of RA patients on TNFi who also received biologics for type 2 conditions was performed. In addition, data collected included age, sex, type of biologic therapy, incident reported bacterial infections and systemic oral corticosteroid use. Incident bacterial infections were tracked 6 months prior to and 6 months after combination biologic therapy based on clinical progress notes, including emergency and urgent care notes, as well as prescriptions for antibiotics. Hospitalization was tracked based on admission and discharge notes. Corticosteroid use was calculated from a review of dose and duration of electronic prescriptions 6 months prior to and after dual biologic therapy and reported as prednisone equivalents.

The institutional review board at Rochester Regional Health System assessed the study prior to initiation and waived the need for formal review and informed consent given its retrospective nature as a case series. Descriptive statistical analysis of mean with 95% CI and median with interquartile range (IQR) was performed with Excel (Microsoft, Redmond, WA, USA). Both mean and median were presented, as they provide different viewpoints on the central tendency of the data in small cohorts.

## Results

Twelve patients were included in the analysis (Table 1). Eleven (91.7%) were female, with a median age of 50 years (IQR 41.5–66.5). Table 1 presents the demographics, biologics used, number of weeks on each medication and number of weeks the medications overlapped. The mean time of TNFi therapy was 159.2 weeks (95% CI 89.6, 228.8) and the median was 103.0 weeks (IQR 83.0–224.1). The mean time of type 2 biologic therapy was 152.5 weeks (95% CI 66.5, 238.5) and the median was 98.9 weeks (IQR 36.4–230.7). The mean overlap of combination biologic therapy was 83.7 weeks (95% CI 56.0, 111.4) and the median was 92.6 weeks (IQR 36.4–109.8). As shown in Table 1, patients were on conventional DMARDs during the overlap of dual biologics.

**Table 1:** Case characteristics.

Patient	Age (years)	Sex	Ethnicity	TNF inhibitor	Total weeks of use	Allergy/immunology diagnosis	Allergy/immunology biologic	Total weeks of use	Overlap weeks of use	Conventional DMARD use during overlap
1	38	F	Hispanic	Adalimumab/etanercept	134.1 (51.7/82.4)	Chronic idiopathic urticaria/asthma	Omalizumab	113.7	113.7	Sulfasalazine (stopped after <4 weeks), methotrexate (stopped after 18 weeks), hydroxychloroquine (stopped after 41 weeks)
2	47	M	White	Adalimumab	105.0	Chronic idiopathic urticaria	Omalizumab	496.6	105.0	Methotrexate (stopped after <4 weeks), hydroxychloroquine (stopped after 30 weeks)
3	67	F	Hispanic	Golimumab	409.6	Asthma	Mepolizumab	31.1	31.1	Hydroxychloroquine (stopped after 15 weeks)
4	69	F	White	Adalimumab	83.4	Asthma	Benralizumab/dupilumab	275.4 (253/22.4)	105.8 (83.4/22.4)	Leflunomide
5	77	F	White	Etanercept	347.6	Atopic dermatitis	Dupilumab	113.7	113.7	Hydroxychloroquine
6	60	F	White	Infliximab	138.0	Asthma	Dupilumab	84.1	84.1	Methotrexate, hydroxychloroquine
7	53	F	White	Golimumab	310.3	Chronic idiopathic urticaria	Omalizumab	186.0	186.0	Hydroxychloroquine
8	39	F	Pakistani	Adalimumab	101.0	Chronic idiopathic urticaria	Omalizumab	365.1	101.0	Hydroxychloroquine
9	44	F	African American	Etanercept	64.7	Asthma	Tezepelumab	27.4	27.4	Sulfasalazine
10	66	F	White	Adalimumab	82.6	Asthma	Tezepelumab	82.6	82.6	Methotrexate
11	46	F	Hispanic	Etanercept	43.4	Chronic idiopathic urticaria	Omalizumab	12.6	12.6	Hydroxychloroquine
12	37	F	White	Adalimumab	90.3	Chronic idiopathic urticaria	Omalizumab	41.7	41.7	Hydroxychloroquine

**Figure 1.**
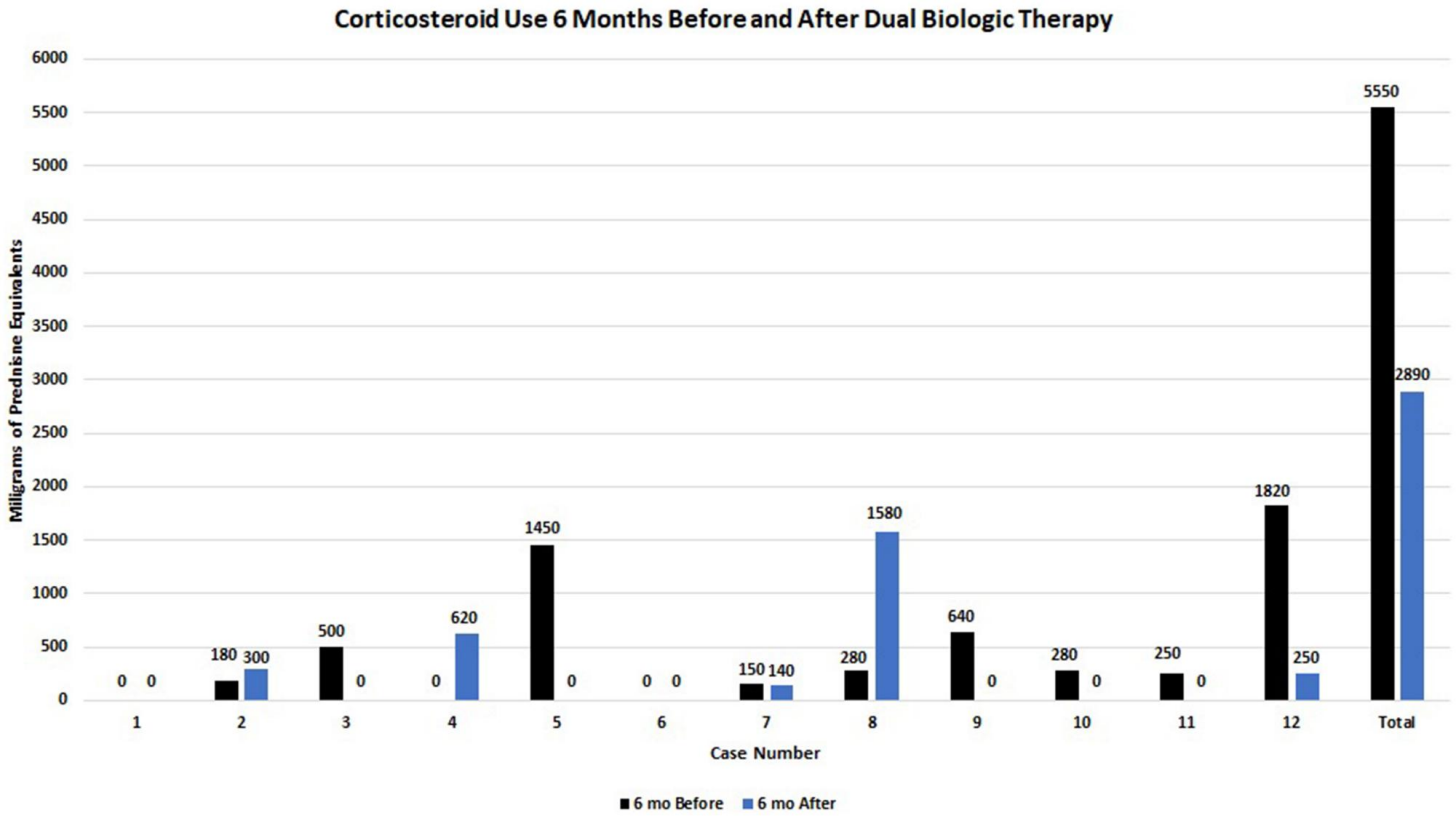
Case-by-case use of oral corticosteroid medications 6 months before and 6 months after starting dual biologic therapy. Corticosteroid cumulative doses are shown in milligrams of prednisone equivalents. Overall, total corticosteroid use was less in the cohort as a whole 6 months after initiating dual biologic therapy

Case-by-case corticosteroid cumulative doses 6 months prior to and 6 months after dual biologic therapy are shown in Fig. 1. The mean corticosteroid cumulative dose 6 months prior to dual biologic agents was 463 mg prednisone equivalent (95% CI 131, 795) and the median was 265 mg (IQR 75–570). The mean corticosteroid cumulative dose 6 months after dual biologic agents was 241 mg prednisone equivalent (95% CI −21, 503) and the median was 0 mg (IQR 0–275). The reasons for corticosteroid use 6 months prior to dual biologic therapy were RA exacerbation (patients 1, 7, 11 and 12), asthma exacerbation (patients 3, 9 and 10), AD exacerbation (patient 5) and urticaria exacerbation (patient 8). The reasons for corticosteroid use 6 months after dual biologic therapy were RA exacerbation (patient 8), asthma exacerbation (patient 4), AD exacerbation (patient 7), urticaria exacerbation (patients 2 and 8) and bronchitis (patient 12).

Supplementary Table S1 shows which bacterial infections occurred in each case 6 months prior to and 6 months after dual biologic therapy. Six bacterial infections occurred prior to combination biologic therapy compared with five after initiating dual biologics. No patient had more than one bacterial infection while on combination biologic therapy. Prior to dual biologics, antibiotics were prescribed six times for four different patients. After dual biologic therapy, four patients were successfully treated with outpatient oral antibiotics and one patient was hospitalized for community-acquired pneumonia requiring intravenous antibiotics.

## Discussion

This case series is the largest to date on RA patients treated with TNFi and a second biologic agent for type 2 inflammation. Our data suggest that combination biologic therapy is associated with decreasing use of corticosteroids without increased rates of incident bacterial infections or antibiotic use over 6 months of combination use.

There is clinical concern regarding infection risk with dual biologics, which may impact decision-making upon starting a biologic medication for a type 2 inflammatory condition in a patient with RA who is already on a TNFi. TNFis are associated with a 20% increased risk of infection, and of those infections, there is a 40% increased risk of serious infection [1]. However, targeting type 2 inflammation is unlikely to increase the risk of infection, as these types of biologics do not impact humoral immunity, as evidenced by maintained vaccine response [4]. Therefore, although the decision to use two biologics should be discussed on a case-by-case basis, this approach should not increase individual side-effect profiles, and this is supported by our data. Importantly, along with a reassuring safety profile, combination biologic therapy was also associated with decreased reliance on corticosteroids. Given the well-known extensive side effects associated with chronic corticosteroid use, combination biologics may be a strategy to improve corticosteroid stewardship [5].

Dual biologic therapy is increasing in numerous conditions, including PsA, IBD and asthma [6, 7]. A recent review of dual biologic therapy for RA revealed heterogeneous findings, with either similar or increased infection rates depending on the agents used, while combination biologic therapy for RA and osteoporosis did not result in increased infection rates [8]. Two single-patient case reports support our conclusion of the safety of combination biologic therapy with a biologic aimed at type 2 inflammation in combination with a TNFi [9, 10]. Similarly, a series of patients on dupilumab plus one of multiple other biologic agents for a variety of conditions, including psoriasis, IBD, chronic spontaneous urticaria and inflammatory arthritis syndromes, also did not find significant infection risks [11]. Most recently, a systemic literature review of 1200 patients who received dual biologic or targeted small molecule agents for multiple refractory type 1 inflammatory conditions, including RA, axial SpA, psoriasis and/or PsA or IBD, was published [12]. While multiple combinations of agents were compared, most combinations exhibited improved clinical benefit with reduced disease activity. While infections did occur, the authors concluded that the safety profile of combination agents was similar to that which had been previously reported without additional signals for worse infection risk [12].

This case series has notable limitations. The small cohort did not allow for appropriate statistical power for comparative statistical analysis and we thus used descriptive analysis. Given the nature of retrospective chart review, despite best efforts, courses of antibiotics or corticosteroids may have been missed, especially if prescribed from out-of-network facilities. We did not follow viral infections, which may often not be documented due to the self-resolving nature of these infections. While no patients specifically sought care for SARS-CoV-2 in chart documentation, it is likely patients contracted this and recovered, as our timeline overlaps with the COVID pandemic. The small cohort limits the ability to generalize conclusions regarding incident infections and should be considered observational. We did not report disease activity scores for RA due to limitations in documentation as well as many patients having multiple scores during follow-up periods of various durations. Statistically assessing and comparing these would be biased. No major adverse events occurred that lead to early discontinuation of the dual biologic agents in this cohort. However, it was not our primary goal to assess for every possible adverse effect, and we urge close clinical monitoring of patients when considering dual biologic agents. While our case series did find overall decreased glucocorticoid use with dual biologic agents, we could not control for individual provider glucocorticoid prescribing habits, and future protocolized studies should be considered.

We conclude from a series of RA patients that combination biologic therapy with TNFi and a biologic aimed at type 2 inflammation for chronic urticaria, asthma and/or AD was not associated with an increased rate of incident infections after 6 months of combination use. Our data also suggest dual biologic therapy may decrease corticosteroid use. However, future clinical trials or prospective large registry data are necessary to calculate the relative risk of complications for dual biologic therapy.

## Supplementary Material

rkaf150_Supplementary_Data

## Data Availability

Data are available upon written request to the corresponding author.
