# Supplementary material for: Combination therapy with TNF inhibitors plus biologics targeting type 2 inflammatory conditions in patients with rheumatoid arthritis: a case series
Source: Rheumatol Adv Pract. 2025 Dec 27;10(1):rkaf150. doi: 10.1093/rap/rkaf150 (PMC12798534; doi:10.1093/rap/rkaf150)
Supplement: rkaf150_Supplementary_Data [file rkaf150_supplementary_data.zip › Supplementary Table S1.docx]

Supplementary Table S1. Infection incidence and type 6 months before and after dual biologic use

| Case | Type of Infection  6 months before dual biologic agents | Antibiotic Use | Type of Infection 6 months after dual biologic agents | Antibiotic use |
| --- | --- | --- | --- | --- |
| 1 | bacterial vaginosis, laryngitis | Yes (2 times) | acute cystitis | yes |
| 2 | none | no | cellulitis | yes |
| 3 | bronchitis | yes | none | no |
| 4 | none | no | pneumonia | yes (hospitalized) |
| 5 | bronchitis, acute conjunctivitis | Yes (2 times) | acute cystitis | yes |
| 6 | none | no | none | no |
| 7 | none | no | none | no |
| 8 | none | no | none | no |
| 9 | acute maxillary sinusitis | yes | none | no |
| 10 | none | no | none | no |
| 11 | none | no | none | no |
| 12 | none | no | bronchitis | yes |
| Total |  | yes = 6 |  | yes = 5 |
